# Mind–Body (Baduanjin) Exercise Prescription for Chronic Obstructive Pulmonary Disease: A Systematic Review with Meta-Analysis

**DOI:** 10.3390/ijerph15091830

**Published:** 2018-08-24

**Authors:** Shi-Jie Liu, Zhanbing Ren, Lin Wang, Gao-Xia Wei, Liye Zou

**Affiliations:** 1 Department of Physical Education, Wuhan University of Technology, Wuhan 430070, China; liushijie0411@whut.edu.cn (S.-J.L.); wanglin123@126.com (L.W.); 2 Department of Physical Education, Shenzhen University, Shenzhen 518060, China; rzb@szu.edu.cn; 3 Key Laboratory of Behavioral Science, Institute of Psychology, Chinese Academy of Sciences, Beijing 100101, China; weigx@psych.ac.cn; 4 Department of Sports Science and Physical Education, The Chinese University of Hong Kong, Shatin, Hong Kong, China

**Keywords:** Baduanjin, Qigong, COPD, rehabilitation

## Abstract

Baduanjin exercise is a traditional Chinese health Qigong routine created by an ancient physician for health promotion. Its mild-to-moderate exercise intensity is suitable for individuals with medical conditions. Recently, a large number of trials have been conducted to investigate the effects of Baduanjin exercise in patients with chronic obstructive pulmonary disease (COPD). It remains to be determined whether Baduanjin exercise prescription is beneficial for the management of COPD patients. Thus, we conducted a systematic review to objectively evaluate the existing literature on this topic. We searched six databases (PubMed, Web of Science, Cochrane Library, Scopus, China National Knowledge Infrastructure, and Wanfang) from inception until early May 2018. The adapted Physical Therapy Evidence Database (PEDro) scale was used for study quality assessment of all randomized controlled trials (RCTs). Based on 95% confidence interval (CI), the pooled effect size (Hedge’s g) of exercise capability (6-Minute Walking Test, 6-MWT), lung function parameters (forced expiratory volume in one second, FEV_1_; forced volume vital capacity, FVC; FEV_1_/FVC ratio), and quality of life were calculated based on the random-effects model. Twenty RCTs (*n* = 1975 COPD patients) were included in this review, with sum scores of the adapted PEDro scale between 5 and 9. Study results of the meta-analysis indicate that Baduanjin is effective in improving exercise capability (Hedge’s g = 0.69, CI 0.44 to 0.94, *p* < 0.001, *I*^2^ = 66%), FEV_1_ (Hedge’s g = 0.47, CI 0.22 to 0.73, *p* < 0.001, *I*^2^ = 68.01%), FEV_1_% (Hedge’s g = 0.38, CI 0.21 to 0.56, *p* < 0.001, *I*^2^ = 54.74%), FVC (Hedge’s g = 0.39, CI 0.22 to 0.56, *p* < 0.001, *I*^2^ = 14.57%), FEV_1_/FVC (Hedge’s g = 0.5, CI 0.33 to 0.68, *p* < 0.001, *I*^2^ = 53.49%), and the quality of life of COPD patients (Hedge’s g = −0.45, CI −0.77 to −0.12, *p* < 0.05, *I*^2^ = 77.02%), as compared to control groups. Baduanjin exercise as an adjunctive treatment may potentially improve exercise capability and pulmonary function of COPD patients as well as quality of life. Baduanjin exercise could be tentatively prescribed for COPD in combination with the conventional rehabilitation program to quicken the process of recovery. To confirm the positive effects of Baduanjin exercise for COPD patients, future researchers need to consider our suggestions mentioned in this article.

## 1. Introduction

Chronic obstructive pulmonary disease (COPD) is a progressive, non-curable disease that causes airflow blockage of lung with breathing-related problems (e.g., dyspnea, wheezing, chest tightness, and frequent coughing with excessive mucus production) [1]. Individuals who suffer COPD have serious complications, including respiratory infection [2], heart problems [3], a higher risk for developing lung cancer [4], pulmonary hypertension [5], and depression [6]. According to the World Health Organization (WHO), the number of individuals with moderate to severe COPD reached roughly 200 million in 2010, accounting for 10 percent of total disabilityand deaths worldwide [7]. It is estimated that by the year 2030 the number of deaths caused by COPD will increase, ranking third in terms of all-cause mortality [8,9,10].

Given that COPD has become one of the most serious public health issues, more and more clinicians and researchers are aware of the importance of pulmonary rehabilitation for COPD patients [11,12]. Of note, exercise as one of the rehabilitation modalities has been gradually recognized to be effective for symptomatic management of COPD, specifically alleviating the clinical symptoms, preventing the exacerbation of lung functions, and improving quality of life of COPD patients [13]. Baduanjin exercise is an ancient Chinese health Qigong exercise [14,15,16] which involves training of musculoskeletal relaxation and stretching, breathing control, and mental focus at a slow pace [17,18]. Because Baduanjin exercise is a mild-to-moderate intensity form of exercise and consists of eight simple movements [19], it has been commonly prescribed for patients with chronic diseases who have low exercise tolerance in Chinese medicine hospitals [20]. Several systematic reviews with meta-analyses of randomized controlled trials indicate the therapeutic effects of Baduanjin exercise for patients with stroke [21], sleep disturbance and musculoskeletal pain [22,23], depression and anxiety [24].

Likewise, since the Chinese Health Qigong Association was officially established in 2011, Baduanjin Qigong as exercise prescription has become more popular and has also been employed [25], for COPD patients in recent years. In particular, a great number of clinical trials have been conducted to investigate the therapeutic effects of the Baduanjin exercise program for COPD patients [26,27,28]. As these studies vary greatly in sample size, study setting, intervention duration, and weekly training dosage, and measuring outcome, it remains largely elusive as to whether Baduanjin exercise is beneficial for COPD patients. Thus, we conducted a systematic review to objectively evaluate the existing literature regarding the effects of Baduanjin exercise for symptomatic management of COPD patients. In this way, study results of this review could provide clinicians with another option to incorporate Baduanjin exercise into the rehabilitation regime of COPD patients, leading to better quality of life.

## 2. Methods

### 2.1. Data Sources

We searched six databases (PubMed, Web of Science, Scopus, China National Knowledge Infrastructure, Wanfang, Cochrane Library) from inception till early May 2018. Keywords were entered as follows: (1) Baduanjin, eight-section Brocade, traditional health Qigong, setting-up exercise; (2) COPD or chronic obstructive pulmonary disease. A cross-reference search was also used to manually identify relevant studies through reference lists of the articles identified at the initial stage. Detailed information of this systematic review and meta-analysis is reported following the Preferred Repointing items for Systematic Reviews and Meta-Analyses (PRISMA) guidelines [29].

### 2.2. Inclusion Criteria and Study Selection

To be included in this review, potentially relevant studies needed to meet the following inclusion criteria: (1) randomized controlled trials (RCTs); (2) included individuals diagnosed with COPD; (3) Baduanjin exercise used as a primary intervention component and comparator with or without other treatments; and (4) a minimum of one outcome (clinical symptoms or health-related parameters) with quantitative data for calculating pooled effect size. Studies that did not meet the above-mentioned requirements were excluded, for example studies with case–control with no randomization, cross-sectional studies, a single-group study with pre-test/post-test design, and reviews. An initial screening was performed by the first author (Shi-Jie Liu) to remove the obviously irrelevant documents. The remaining of possibly relevant articles were further evaluated by two independent reviewers (Shi-Jie Liu and Liye Zou) to confirm their eligibility. When any disagreement between the two review authors occurred, a third review author (Lin Wang) was invited to verify the eligibility of the uncertain article by discussion with them.

### 2.3. Study Quality Assessment for Eligible Studies Selected

To assess the study quality of studies selected, the Physical Therapy Evidence Database (PEDro) scale was adopted [30]. The original 11-item PEDro scale involves evaluation of eligibility criteria, random assignment, allocation concealment, baseline equivalence, blinding of stakeholders (participants, instructors, and assessors), retention rate of 85% and above, intention-treat-analysis, between-group statistical comparisons, and point measurement and measurement variability. Given the fact that the blinding of participants and instructors is impossible during exercise intervention, these two items were not considered for computing sum scores of methodological quality of each individual eligible study. Since all participants in the eligible studies had to be diagnosed with COPD for inclusion of this review, the eligibility criteria of all studies selected were satisfied; thus this item was not considered, and one point was removed from the sum scores. Furthermore, Baduanjin exercise as a primary intervention is possibly combined with other treatment(s) like drug therapy or usual care, such this method is ethically reasonable but could affect interpretation of study findings. Thus, “isolated Baduanjin intervention” as a new item was added, which ultimately resulted in a total of 9 items and each item was worth one point [31].

### 2.4. Data Extraction and Synthesis

Data extraction was independently carried out by two review authors (Shi-Jie Liu and Liye Zou) and a standardized table is used. Features of all RCTs selected are extracted, including reference (author and year of publication), location and language of publication, characteristics of patients (sample size and attrition rate, mean age/age range, and course of disease), intervention protocol (weekly training dosage and duration), measuring outcome and measurement, and adverse event and follow-up assessment. To calculate pooled effect size (Hedge’s g, with small effect = 0.2, medium effect = 0.5, and large effect = 0.8), we used Comprehensive Meta-Analysis Software (Bio. Stat. Inc., Englewood, NJ, USA) based on the number of participants of each group and its quantitative data (mean and standard deviation) at baseline and post-intervention [32]. Given a difference in sample size, testing instrument, and course of disease, the random-effects model was used. The value of *I*^2^ was used to determine whether heterogeneity (small = 25%, moderate = 50%, and large = 75%) across the selected studies existed. Finally, we used the funnel plot and Egger’s regression intercept test to detect publication bias. Based on the visual inspection and data, outlying studies were removed so that symmetrical funnel plot and without statistical significance were observed.

## 3. Results

### 3.1. Study Selection

**Figure 1:**
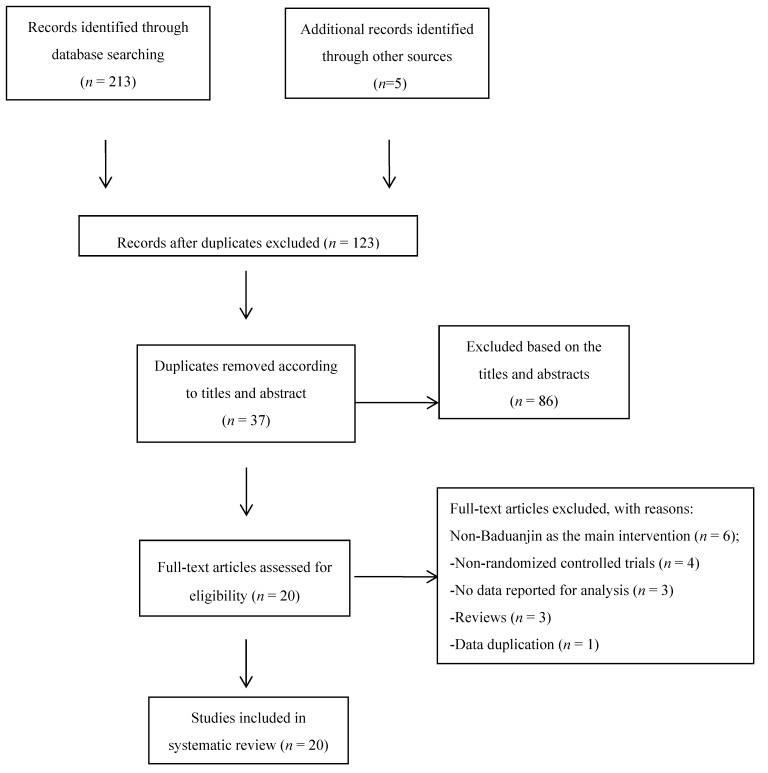
Flow of study selection.

The procedures of study selection are shown in Figure 1. The electronic and manual searches resulted in 218 articles. Based on the titles of initially identified articles, there were 95 duplicates and therefore they were removed. After looking through the abstract of remaining articles, 86 irrelevant documents were excluded and 37 articles remained. Furthermore, 17 documents were excluded because they were non-randomized controlled trials (*n* = 4), review studies (*n* = 3), had a main intervention other than Baduanjin (*n* = 6), had data duplication (*n* = 1), or had no data reported for analysis (*n* = 3) (Figure 1). This left only 20 eligible articles to be included for the meta-analysis, including 18 in Chinese and 2 in English.

### 3.2. Study Characteristics

The characteristics of the 20 studies are presented in Table 1. These studies were published between 2009 and 2018. In total, there were 1975 COPD patients, sample size across studies ranged from 24 to 320, with age range from 59.67 to 73.12. The average duration of the course of the disease was 1.64 to 16.21 years. Disease severity ranged from mild to very severe, as reported by study authors: four studies [33,34,35,36] recruited participants with disease severity ranging from mild to severe, and two studies [37,38] recruited patients with moderate disease, whereas other studies did not specify the severity. Unsurprisingly, Baduanjin training was integrated with either drug therapy [37,38,39,40,41,42,43,44,45] or usual care [34,35,42,46,47,48]. Baduanjin training was also integrated with convention therapy [45,38] and breathing technique training [33]. Only two studies [49,50] used isolated Baduanjin training for comparison with other control conditions. The intervention duration in Baduanjin training varied greatly across studies selected, ranging from 3 to 12 months. The frequency of training ranged from three to seven sessions each week, with each session lasting 30 to 60 min.

### 3.3. Methodological Quality

Based on the adapted PEDro scale (Table 2), methodological quality of all eligible RCTs were scored, ranging from 5 to 9. Allocation concealment [33,34,36,40,41,42,43,44,46,47,48,52], blinding of assessors [33,34,35,36,37,38,39,40,41,42,43,44,45,47,48,51,52], and isolated Baduanjin exercise interventions [33,34,35,36,37,38,39,40,41,42,43,44,45,46,47,48,51,52] were absent. Intention-to-treat analysis for missing data was not employed in three studies [33,37,47]. One study [49] reported a greater than 15% attrition rate, along with the unclear description of point measure and measures of variability.

### 3.4. Effects of Baduanjin on 6-Minute Walking Test

**Figure 2:**
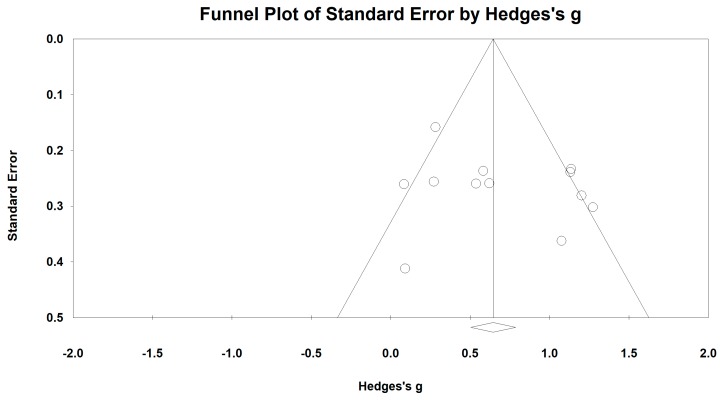
Funnel plot of publication bias for the 6-Minute Distance Test.

**Figure 3:**
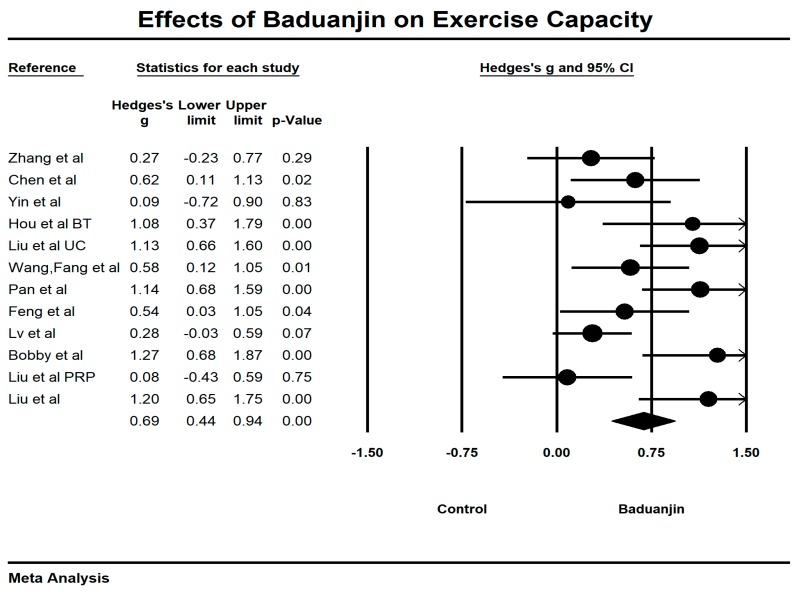
Effects of Baduanjin on exercise capability measured by the 6-MWT (BT = breathing technique; PRP = conventional pulmonary rehabilitation; UC = usual care).

Fourteen studies examined the effects of Baduanjin exercise on the 6-MWT (longer distance indicates better exercise capability). Four outlying studies [33,38,44,45] were visually detected through the Funnel Plot along with the Egger’s Regression Test. After removing these outliers, symmetrical funnel plot (Figure 2) was observed (Egger’s regression intercept = 2.156, *p* = 0.339). The meta-analysis of the 10 RCTs has shown that Baduanjin training was effective in improving performance of the 6-MWT (Hedge’s g = 0.69, CI 0.44 to 0.94, *p* < 0.001, *I*^2^ = 66%; Figure 3), as compared to control groups.

### 3.5. Effects of Baduanjin on Lung Functions

**Figure 4:**
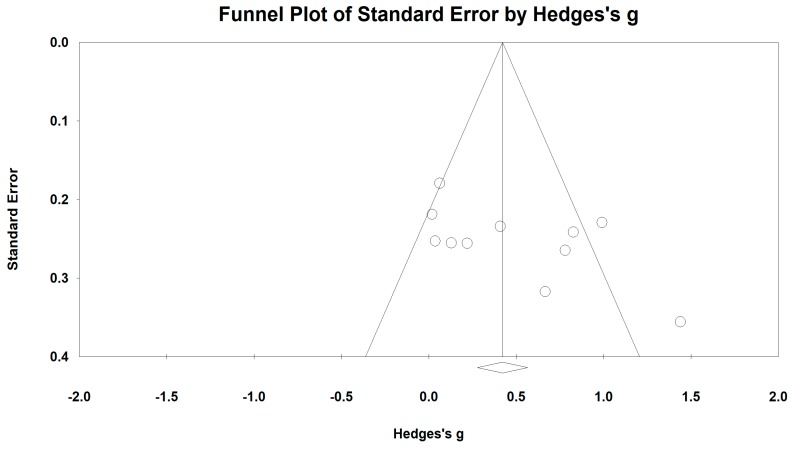
Funnel plot of publication for FEV_1_.

**Figure 5:**
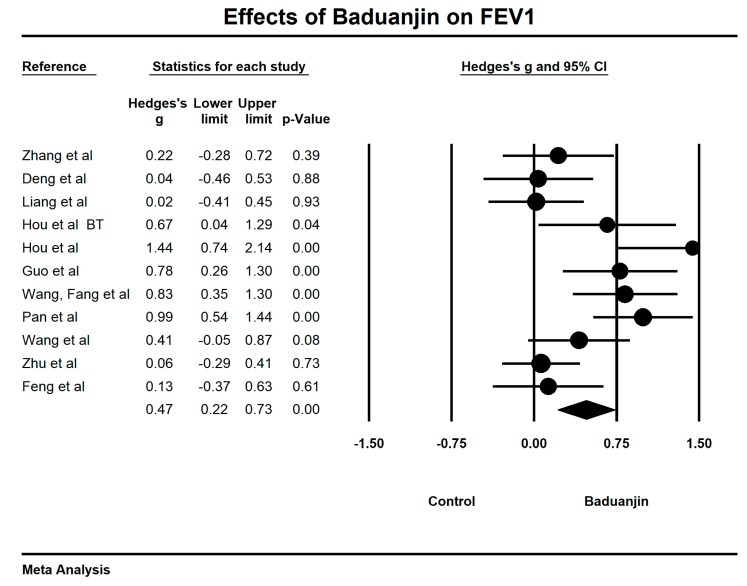
Effect of Baduanjin on FEV_1_ (BT = breathing technique training).

For the forced expiratory volume in one second (FEV_1_), we used the funnel plot to visually detect publication bias and one outlying study [42] was removed, leading to a non-significant symmetry (Egger’s regression intercept = 5.81, *p* = 0.062; Figure 4). A meta-analysis of the 10 remaining RCTs showed that Baduanjin was effective in improving FEV_1_ (Hedge’s g = 0.47, CI 0.22 to 0.73, *p* < 0.001, *I*^2^ = 68.01%; Figure 5), as compared to control groups (Table 3).

**Figure 6:**
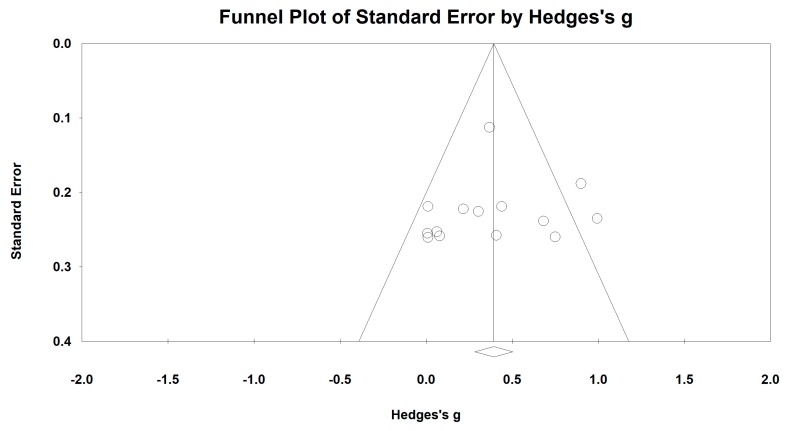
Funnel plot of publication bias for FEV_1_%.

**Figure 7:**
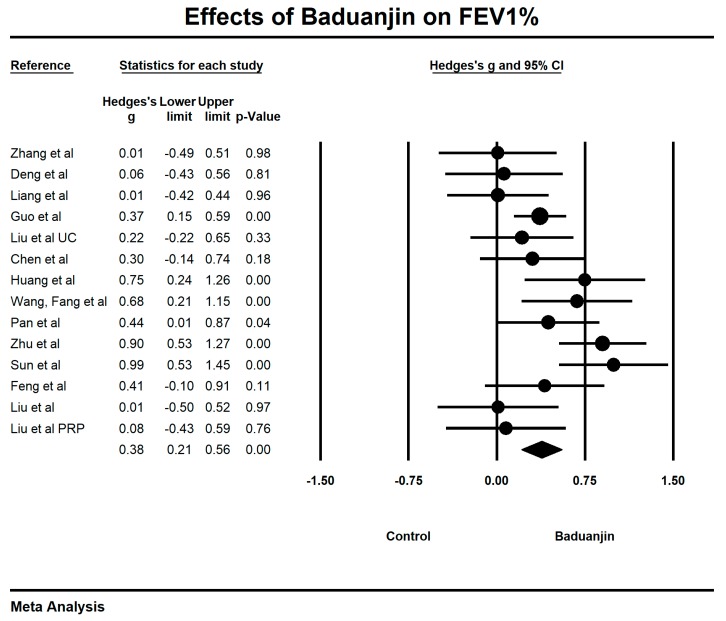
Effects of Baduanjin on FEV1% (PRP = conventional-pulmonary rehabilitation programs; UC = usual care).

When we performed the meta-analysis regarding FEV_1_%, we used the funnel plot to visually detect two outlying studies [40,44] and they were removed, leading to a non-significant symmetry (Egger’s regression intercept = −0.57, *p* = 0.719; Figure 6). The meta-analysis of the 14 remaining RCTs showed that Baduanjin was effective in improving FEV_1_% (Hedge’s g = 0.38, CI 0.21 to 0.56, *p* < 0.001, *I*^2^ = 54.74%; Figure 7).

**Figure 8:**
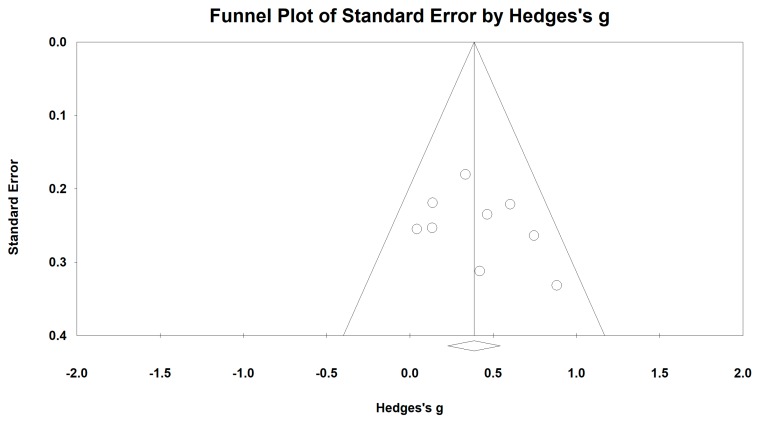
Funnel plot of publication bias for FVC.

**Figure 9:**
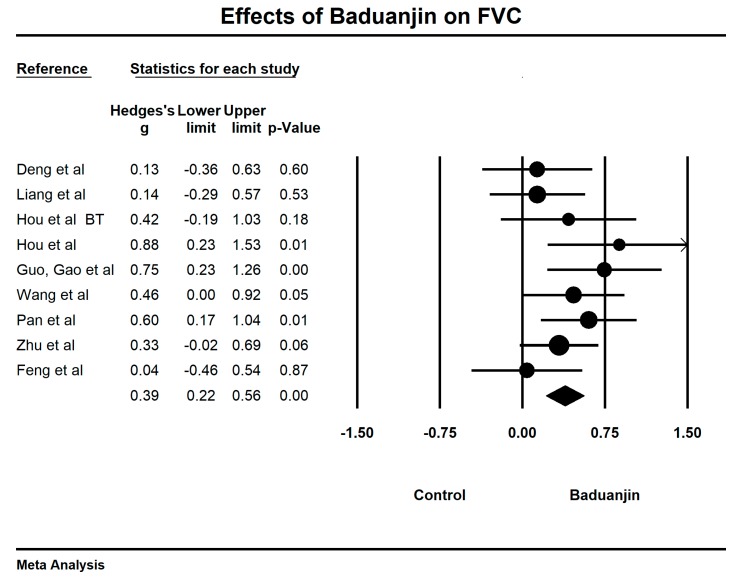
Effects of Baduanjin on FVC (BT = breathing technique training).

When we performed the meta-analysis regarding forced volume vital capacity (FVC), we used the funnel plot to visually detect one outlying study [39] and then it was removed, leading to a non-significant symmetry (Egger’s regression intercept = 2.022, *p* = 0.367; Figure 8). The meta-analysis of the eight RCTs showed that Baduanjin was effective in improving FVC (Hedge’s g = 0.39, CI 0.22 to 0.56, *p* < 0.001, *I*^2^ = 14.57%; Figure 9).

**Figure 10:**
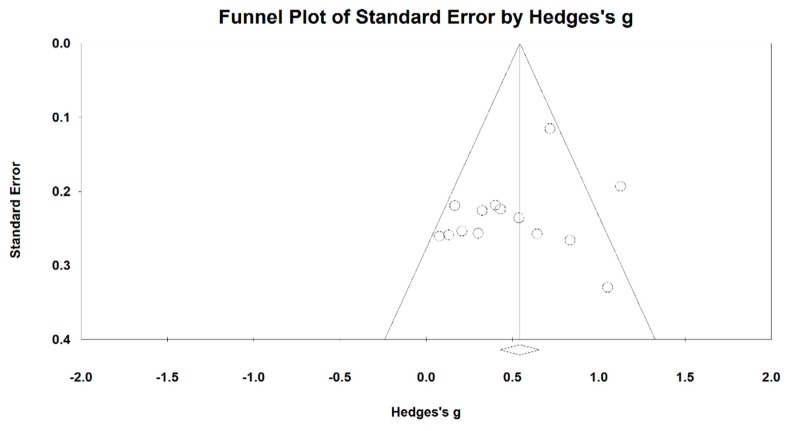
Funnel plot of publication bias for FEV_1_/FVC%.

**Figure 11:**
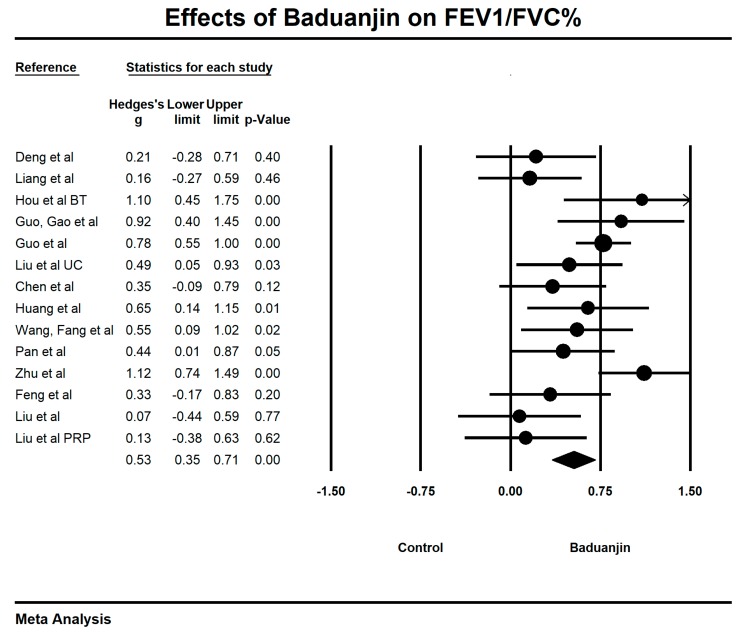
Effects of Baduanjin on FEV_1_/FVC% (BT = breathing technique training; UC = usual care).

When we performed the meta-analysis regarding FEV_1_/FVC%, we used the funnel plot to visually detect one outlying study [33] and then it was removed, leading to a non-significant symmetry (Egger’s regression intercept = −1.737, *p* = 0.23; Figure 10). The meta-analysis of the 13 remaining RCTs showed that Baduanjin was effective in improving FEV_1_/FVC% (Hedge’s g = 0.53, CI 0.35 to 0.71, *p* < 0.001, *I*^2^ = 53.49%; Figure 11).

### 3.6. Effects of Baduanjin on Quality of Life

**Figure 12:**
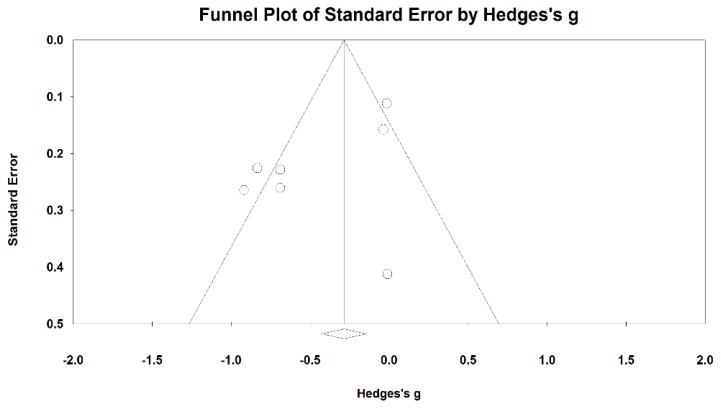
Funnel plot of publication bias for quality of life.

**Figure 13:**
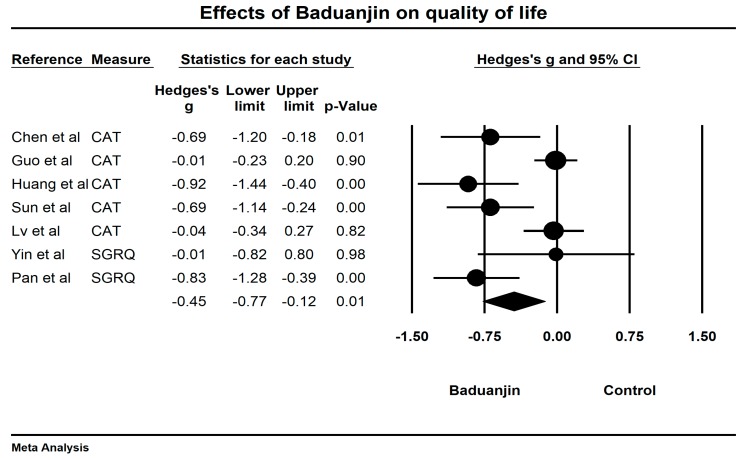
Effects of Baduanjin on quality of life of COPD patients.

Both the SGBQ and the CAT were used to evaluate quality of life, with lower sum scores indicating better quality of life. Given that two studies [33,43] did not report the sum scores of the SGRQ, in total 7 RCTs [36,37,38,41,42,48,52] were included for meta-analysis. As a symmetrical funnel plot was visually observed along with a non-significant value (Egger’s regression intercept = −3.479, *p* = 0.094; Figure 12), the study findings of the seven studies included for meta-analysis showed that Baduanjin was effective in improving the quality of life of COPD patients (Hedge’s g = −0.45, CI −0.77 to −0.12, *p* < 0.05, *I*^2^ = 77.02%; Figure 13).

## 4. Discussion

To the best of our knowledge, this is the first systematic review with meta-analytical method to objectively evaluate the therapeutic effects of Baduanjin training for COPD patients. Study findings of the current review indicate that Baduanjin exercise as an adjunctive treatment in the management of COPD patients may have the potential to improve exercise capability, lung function, and quality of life. All eligible RCTs were published between 2009 and 2018, suggesting that it is a newly expanding research field.

Exercise capability and lung function of COPD patients gradually declines, which can prevent them from participating in physical activities or even activities of daily living. Sedentary lifestyle and physical inactivity would worsen these physical functions, leading to reduced quality of life [53]. To escape this vicious circle, a mild-to-moderate aerobic exercise program should be prescribed for pulmonary rehabilitation of COPD patients. The purpose of this current review was to evaluate the efficacy and safety of Baduanjin exercise training in the symptomatic management of COPD patients. No adverse events occurred in any of studies, and the positive findings in the current review are in line with previous meta-analyses of randomized controlled trials investigating the beneficial effects of other mind–body exercises (Tai Chi, Yoga, and Qigong) for COPD patients [54,55,56].

Overall, improved exercise capability and lung function as well as the quality of life of COPD patients may be due to movement patterns of Baduanjin routine. First, a slow-paced Baduanjin routine as a mild-to-moderate intensity aerobic exercise is presumably suitable for COPD patients who have low exercise tolerance [20]. Furthermore, training with a Baduanjin routine involves musculoskeletal stretching and relaxation, diaphragmatic breathing (inhaling and exhaling through your nose), and mental concentration in a coordinated way. These components of movements may be the key to strengthening the lung capability and diaphragm of COPD patients. For instance, Movement 1 (Two Hands Hold up the Heavens) and 2 (Drawing the Bow to Shoot the Eagle) in the Baduanjin routine involve a large amount of upper-limb stretching. This musculoskeletal stretching training in the upper body can expand the thoracic, diaphragmatic, and abdominal muscles. Meanwhile, respiratory muscles are trained and the contraction force is enhanced to concentrate on the stretching of the patient’s trunk and limbs, providing the opportunity to potentially strengthen muscle and improve limb coordination and exercise performance. In addition, mental focus and relaxation are also integrated in Baduanjin training where COPD patients may feel less fatigue and a more pleasant sensation, which may build better mental health and increase adherence to this exercise protocol. As exercise capability and lung function improve, COPD patients naturally experience better quality of life.

While the overall results of meta-analysis are in supportive of the effect of Baduanjin training on improving exercise capability of COPD, the positive effect in some studies [37,39,50,52] did not reach statistical significance, and as such should be interpreted with caution. Specifically, this occurred in two studies where Baduanjin training was compared with active controls: one with breathing technique training [37] and another with conventional pulmonary rehabilitation program (walking plus ball exercise) [50]. Baduanjin training is possibly comparable to these two research-based rehabilitation methods [57], so it is reasonable to observe non-statistical significance in these two comparisons. In addition, an intervention duration of less than 6 months was applied in other two studies [39,52] in which a trend of increasing exercise capability after Baduanjin training was observed, but it was not statistically significant. This suggests that Baduanjin training of more than 6 months with an optimal training intensity may be more preferable in this special population.

Impaired lung function (e.g., FEV_1_ and FVC) of COPD patients is commonly recognized. Overall, statistically significant positive effects were observed in these lung function parameters when Baduanjin training as a core intervention component was compared with control groups. Taking a closer look inside the individual studies, statistically significant differences were not reached in some studies in which Baduanjin training was integrated with usual care, drug therapy, conventional therapy, or a breathing technique training for comparison with the active control alone. The synergistic effect between Baduanjin training and drug therapy may produce a Baduanjin–drug interaction, leading to a comparable influence on these parameters. Thus, an independently applied Baduanjin training intervention protocol should be considered in future studies. Similarly, this synergistic effect existed in those studies where the outcome of “quality of life” was meta-analyzed.

## 5. Limitations

Study limitations in this current review need to be pointed out. First, the Baduanjin training in 90% of studies selected was integrated with other components, particularly drug therapy and usual care. Undoubtedly, this combination is ethically preferable because drug therapy is a mainstream rehabilitation method. However, must be admitted that the positive effects of Baduanjin training may be due to a synergistic effect and not Baduanjin training alone. Second, the Baduanjin intervention protocol (duration, frequency, and intensity) varied greatly across studies. This could, to greater extent, affect the reliability of the pooled effect sizes and make it difficult for review authors to draw a firm conclusion about the optimal training recommendation. Furthermore, none of studies selected used follow-up measurements, so positive results regarding the long-term effects of Baduanjin training still remain elusive. Third, different styles (standing or sitting) of the Baduanjin routine or individual movements were applied in these intervention studies; these factors may exaggerate the statistical heterogeneity of the pooled results. For the posture of Baduanjin can refer to the Supplementary Materials. Positive results from most of studies selected with high risk of bias (lack of allocation concealment, blinded assessors, intention-to-treat analysis, and isolated Baduanjin training) should be extrapolated with caution. Finally, study participants recruited in the studies selected were Chinese (mainland and Hong Kong). It is unknown whether the similar results regarding the positive effect of Baduanjin training in COPD patients of other races would be observed.

## 6. Conclusions

Study results of this current review indicate that Baduanjin training as an adjunctive treatment may potentially improve exercise capability and pulmonary function of COPD patients as well as quality of life. Baduanjin training could be tentatively prescribed for COPD in combination with conventional rehabilitation programs to quicken the process of recovery. To confirm the positive effects of Baduanjin training for COPD patients, future researchers need to consider our suggestions as mentioned above.

## Figures and Tables

**Table 1:** Summary of randomized controlled trials.

Reference	Location (Language)	Participant Characteristics			Intervention Program	Baduanjin Training			Outcome Measured	Adverse Event; Follow-Up
		Sample Size (Attrition Rate)	Mean Age or Age Range	Course of Disease		Frequency (weekly)	Time (min)	Duration (week)		
Zhang et al. [39]	Changchun, China (Chinese)	60 (0%)	BJ: 68.50 (9.18) CG: 68.03 (7.18)	NR	BJ: Baduanjin + Drug Therapy CG: Drug Therapy	7	/	8	Lung function (FEV_1_, FEV_1_%, FVC), Exercise Capacity (6-MWT)	No; No
Deng et al. [46]	Fujian, China (Chinese)	60 (1.6%)	BJ: 66.26 (5.13) CG: 66.90 (4.63)	BJ: 4.68 (2.54) CG: 4.77 (2.52)	BJ: Baduanjin + Usual Care CG: Usual Care	7	30	12	Lung function (FEV_1_, FEV_1_%, FVC, FEV_1_/FVC)	No; No
Liang et al. [47]	Guangdong, China (Chinese)	82 (0%)	BJ: 60.23 (9.32) CG: 60.23 (9.32)	BJ: 4.25 (2.05) CG: 4.25 (2.05)	BJ: Baduanjin + Usual Care CG: Usual Care	7	30	12	Lung function (FEV_1_, FEV_1_%, FVC, FEV_1_/FVC)	No; No
Chen et al. [48]	Fujian, China (Chinese)	60 (3.4%)	BJ: 66.26 (5.13) CG: 66.90 (4.63)	BJ: 4.68 (2.54) CG: 4.77 (2.52)	BJ: Baduanjin + Usual Care CG: Usual Care	7	30	12	Exercise Capacity (6-MWT), Quality of life (CAT)	No; No
Yin et al. [37]	Guangzhou, China (Chinese)	24 (0%)	BJ: 70.40 (7.56) CG: 69.17 (7.66)	NR	BJ: Baduanjin + Drug Therapy CG: Breathing training + Drug Therapy	7	30	24	Quality of life (SGRQ), Exercise Capacity (6-MWT)	No; No
Hou et al. [33]	Dongwan, China (Chinese)	60 (0%)	BJ: 63.34 (5.95) CG1: 62.87 (6.01) CG2: 63.77 (6.14)	NR	BJ: Baduanjin + Breathing Training CG1: Breathing Training CG2: Unaltered Lifestyle	2	30	12	Lung function (FEV_1_, FVC, FEV_1_/FVC), Exercise Capacity (6-MWT), Quality of life (SGRQ)	No; No
Guo, Gao et al. [40]	Qingdao, China (Chinese)	60 (0%)	BJ: 63.4 (NR) CG: 62.8 (NR)	BJ: 4.67 (1.54) CG: 4.75 (1.52)	BJ: Baduanjin + Drug Therapy CG: Drug Therapy	>4	30	24	Lung function (FEV_1_, FEV_1_%, FVC, FEV_1_/FVC)	No; No
Guo et al. [41]	Qingdao, China (Chinese)	320 (0%)	BJ: 64.87 (8.86) CG: 64.15 (8.97)	BJ: 16.19 (5.48) CG: 16.21 (5.53)	BJ: Baduanjin + Drug Therapy CG: Drug Therapy	>4	30	24	Lung function (FEV_1_%, FEV_1_/FVC), Quality of life (CAT)	No; No
Liu et al. [34]	Chengdu, China (Chinese)	80 (0%)	BJ: 59.77 (7.08) CG: 60.67 (6.95)	NR	BJ: Baduanjin + Usual Care CG: Usual Care	7	30	12	Lung function (FEV_1_%, FEV_1_/FVC), Exercise Capacity (6-MWT)	No; No
Chen, Liu et al. [35]	Chengdu, China (Chinese)	78 (0%)	BJ: 60.52 (7.24) CG: 59.67 (6.91)	NR	BJ: Baduanjin + Usual Care CG: Usual Care	7	30	12	Lung function (FEV_1_%, FEV_1_/FVC)	No; No
Huang et al. [42]	Nanjing, China (Chinese)	62 (0%)	BJ: 68.24 (3.28) CG: 69.77 (4.42)	BJ: 24.60 (10.6) CG: 17.32 (14.44)	BJ: Baduanjin + Drug Therapy CG: Drug Therapy	7	30	24	Lung function (FEV_1_, FEV_1_%, FEV1/FVC) Quality of life (CAT)	No; No
Wang, Fang et al. [43]	Dalian, China (Chinese)	73 (3.9%)	BJ: 63.17 (9.95) CG: 63.67 (9.75)	BJ: 15.17 (6.73) CG: 14.83 (7.89)	BJ: Baduanjin + Drug Therapy CG: Drug Therapy	7	30	12	Lung function (FEV_1,_ FEV_1_%, FVC, FEV_1_/FVC), Exercise Capacity (6-MWT), Quality of life (SGRQ)	No; No
Pan et al. [36]	SiChuan, China (Chinese)	82 (0%)	BJ: 60.7 (5.6) CG: 61.8 (7.2)	BJ: 6.7 (6.2) CG: 8.8 (5.3)	BJ: Baduanjin + Usual Care CG: Usual Care	7	30	24	Lung function (FEV_1_, FEV_1_%, FVC, FEV_1_/FVC), Exercise Capacity (6-MWT), Quality of life (SGRQ)	No; No
Wang [44]	Beijing, China (Chinese)	73 (0%)	BJ: 66.76 (5.80) CG: 66.69 (4.60)	BJ: 11.02 (3.38) CG: 10.85 (3.53)	BJ: Baduanjin + Drug Therapy CG: Drug Therapy	7	30	48	Lung function (FEV_1_%, FEV_1_), Exercise Capacity (6-MWT)	No; No
Zhu et a. [45]	ChangSha, China (Chinese)	125 (0%)	BJ: 69.00 (8.70) CG: 68.00 (9.20)	BJ: 12.50 (10.70) CG: 10.80 (8.90)	BJ: Baduanjin + Drug Therapy CG: Drug Therapy	14	30	24	Lung function (FEV_1_, FEV_1_%, FVC, FEV_1_/FVC), Exercise Capacity (6-MWT)	No; No
Sun et al. [38]	ChangChun, China (Chinese)	60 (5.4%)	BJ: 62.97 (6.87) CG: 63.21 (7.02)	BJ: 11.02 (3.38) CG: 10.85 (3.53)	BJ: Baduanjin + Drug Therapy CG: Drug Therapy	7	30	48	Lung function (FEV1%) , Exercise Capacity (6-MWT) and Quality of life (CAT)	No; No
Feng et al. [51]	GuangZhou, China (Chinese)	60 (0%)	BJ: 63 (4.00) CG: 62 (5.00)	BJ: 5.2 (2.2) CG: 5.4 (1.9)	BJ: Baduanjin + Conventional Therapy CG: Conventional Therapy	10	45	24	Lung function (FEV_1_, FEV_1_%, FVC, FEV1/FVC), Exercise Capacity (6-MWT)	No; No
Lv et al. [52]	Beijing, China (Chinese)	160 (0%)	BJ: 64.88 (8.87) CG: 63.14 (9.12)	BJ: 16.21 (5.49) CG: 16.57 (5.17)	BJ: Baduanjin + Conventional Therapy CG: Conventional Therapy	7	20	2	Exercise Capacity (6-MWT), Quality of life (CAT)	No; No
Bobby et al. [49]	Hong kong, China (English)	51 (36.25%)	BJ: 71.75 (1.05) CG: 73.12 (1.33)	NR	BJ: Baduanjin CG: walking training	>4	30	24	Exercise Capacity (6-MWT)	No; No
Liu et al. [50]	ShangHai, China (English)	132 (10.60%)	BJ: 61.82 (7.69) CG1: 62.2 (6.34) CG2: 61.34 (8.34)	BJ: 7.54 (2.73) CG1: 7.75 (2.20) CG2: 6.34 (2.34)	BJ: Baduanjin CG1: Health Education CG2: Conventional Pulmonary Rehabilitation (walking + ball training)	3	60	24	Lung function (FEV_1_%, FEV_1_/FVC), Exercise Capacity (6-MWT)	No; No

Note: BJ = Baduanjin; CG = control group; FEV_1_ = the forced expiratory volume in one second; FEV_1_% = percentage of the forced expiratory volume in one second; FVC = forced vital capacity; FEV_1_/FVC = the amount of air exhaled in the first second divided by all of the air exhaled during a maximal exhalation; 6-MWT = 6-Minute Walking Test; SGRQ = St. George’s Breathing Questionnaire; CAT = COPD (chronic obstructive pulmonary disease) Assessment Test; NR = not reported.

**Table 2:** Study quality assessment for eligible randomized controlled trials.

Author [Reference]	Item 1	Item 2	Item 3	Item 4	Item 5	Item 6	Item 7	Item 8	Item 9	Score
Zhang et al. [39]	1	1	1	0	1	1	1	1	0	7
Deng et al. [46]	1	0	1	1	1	1	1	1	0	7
Liang et al. [47]	1	0	1	0	1	0	1	1	0	5
Chen et al. [48]	1	0	1	0	1	1	1	1	0	6
Yin et al. [37]	1	1	1	0	1	0	1	1	0	6
Hou et al. [33]	1	0	1	0	1	0	1	1	0	5
Guo, Gao et al. [40]	1	0	1	0	1	1	1	1	0	6
Guo et al. [41]	1	0	1	0	1	1	1	1	0	6
Liu et al. [34]	1	0	1	0	1	1	1	1	0	6
Chen, Liu et al. [35]	1	1	1	0	1	1	1	1	0	7
Huang et al. [42]	1	0	1	0	1	1	1	1	0	6
Wang, Fang et al. [43]	1	0	1	0	1	1	1	1	0	6
Pan et al. [36]	1	0	1	0	1	1	1	1	0	6
Wang [44]	1	0	1	0	1	1	1	1	0	6
Zhu et al. [45]	1	1	1	0	1	1	1	1	0	7
Sun et al. [38]	1	1	1	0	1	1	1	1	0	7
Feng et al. [51]	1	1	1	0	1	1	1	1	0	7
Lv et al. [52]	1	0	1	0	1	1	1	1	0	6
Bobby et al. [49]	1	1	1	1	0	1	1	0	1	7
Liu et al. [50]	1	1	1	1	1	1	1	1	1	9

Note: Item 1 = randomization; Item 2 = concealed allocation; Item 3 = similar baseline; Item 4 = blinding of assessors; Item 5 = more than 85% retention; Item 6 = missing data management (intention-to-treat analysis); Item 7 = between-group comparison; Item 8 = point measure and measures of variability; Item 9 = isolated Baduanjin intervention; 1 = explicitly described and present in details; 0 = absent, inadequately described, or unclear.

**Table 3:** Study results of meta-analysis regarding lung function parameters.

Outcomes	Studies	Sample Size		Hedge’s g	95% CI	Heterogeneity	
		Baduanjin Group	Control Group			*I*^2^ (%)	*p* Value
FEV1 value	10	398	411	0.47	0.22–0.73	68.01%	*p* < 0.001
FEV1% value	13	711	706	0.38	0.21–0.56	54.74%	*p* < 0.001
FVC	8	330	344	0.39	0.22–0.56	14.57%	*p* < 0.001
FEV1/FVC value	13	629	655	0.5	0.33–0.68	53.49%	*p* < 0.001

Note: Lung functions consisted of FEV_1_ (forced expiratory volume in one second, *n* = 12 RCTs), FEV_1_% (*n* = 16 RCTs), FVC (forced vital capacity, *n* = 10 RCTs), and FEV_1_/FVC (*n* = 14 RCTs).

## Supplementary Materials

Supplementary data: https://www.youtube.com/watch?v = Q8jsDDJW2y0 (Baduanjin exercise routine).
